# Development and validation of a risk prediction model of delayed onset of lactogenesis among mothers with gestational diabetes mellitus: a prospective multicenter study

**DOI:** 10.3389/fcdhc.2026.1721649

**Published:** 2026-03-26

**Authors:** Yu-Ting Luo, Min Xu, Zhi-Dong Guo, Yun-Xia Liu, Li Liu

**Affiliations:** Nursing Department, The First Affiliated Hospital of Sun Yat-sen University, Guangzhou, China

**Keywords:** delayed onset of lactogenesis, factor, gestational diabetes mellitus, nomograph, risk prediction model

## Abstract

**Objective:**

The aim of this study is to develop and validate a prediction model of delayed onset of lactogenesis among mothers with gestational diabetes mellitus.

**Methods:**

This was a prospective study. A total of 511 mothers with GDM hospitalized at seven tertiary (grade 3A) hospitals in five cities of Guangdong Province, China, between October 10, 2023, and December 8, 2024, were enrolled in the study using convenience sampling. Univariate regression, LASSO regression and logistic regression were used to construct and validation risk prediction model. Logistic regression and R language was used to draw the nomogram. Model performance was evaluated using the area under the receiver operator characteristic curve (AUC), Hosmer-Lemeshow goodness-of fit test, and clinical decision curve analysis, assessing the discrimination ability, calibration, and clinical utility of the model.

**Results:**

Based on logistic regression analysis, we identified several significant delayed onset of lactogenesis risk factors for mothers with gestational diabetes mellitus: pre-delivery BMI, Edinburgh Postnatal Depression Scale score, serum albumin levels, LATCH score, blood glucose control during pregnancy. The AUC was 0.828, (95%*CI*:0.779~0.877),with a sensitivity of 70.9%, a specificity of 83.7%,optimal cut-off point of 0.341, a maximum Youden index of 0.546. The Hosmer-Lemeshow test yielded a *χ*^2^ value of 7.226 and *p* = 0.546.The internal verification showed the AUC was 0.806(95%*CI*:0.728, 0.884), indicating the model’s high discrimination ability. Calibration curves showed good agreement between predicted and observed values, confirming good calibration. The clinical decision curve analysis further supported the model’s clinical utility.

**Conclusion:**

The prediction model constructed and verified in this study was to predict delayed onset of lactogenesis risk for mothers with gestational diabetes mellitus, providing an effective evaluation tool for healthcare professionals.

## 1 Introduction

Gestational diabetes mellitus (GDM) refers to abnormal glucose tolerance with onset or first recognition during pregnancy (1). GDM is one of the most common medical complications of pregnancy (2, 3). According to statistics released by the International Diabetes Federation (IDF), the global prevalence of hyperglycemia in pregnancy reached 16.7% by 2021, with GDM accounting for the majority of cases at a striking proportion of 80.3% (4), indicating that GDM has evolved into a global and public health challenge. China has a notably higher incidence rate. A survey revealed that the prevalence of GDM reached 21% in China in 2020, significantly surpassing the rates in Japan, South Korea and Thailand.

Breast milk provides optimal nutrition for infant growth, making it the ideal food for infants (5). Breastfeeding confers significant advantages, including reducing the risks of breast cancer, ovarian cancer, and type 2 diabetes, as well as helping prevent postpartum depression (6). The longer breastfeeding is sustained, the greater the socioeconomic benefits (7). For many years, the World Health Organization (WHO) and the United Nations Children’s Fund (UNICEF) have jointly developed a series of laws and policies to support breastfeeding (8). However, the current status of breastfeeding remains suboptimal (9).

Delayed onset of lactogenesis (DOL) is defined as the perception of breast fullness and engorgement experienced beyond 72 h postpartum (10). DOL increases the difficulties of early breastfeeding, raising the risk of weaning within 4 weeks postpartum by 60% (11). DOL, a critical factor affecting exclusive breastfeeding, has become a significant barrier to achieving the WHO-recommended 50% exclusive breastfeeding rate within the first six months (12). Studies indicate that mothers with GDM have a 1.84 times higher risk of DOL compared to non-GDM mothers (13). The reported incidence of DOL among GDM mothers ranges from 25.2% to 35.0% (13, 14).

Risk prediction models are widely employed in clinical practice for disease risk assessment and early screening. By modeling probabilistic relationships within data, they generate quantifiable, optimal estimates for future uncertain events. While existing research has primarily focused on identifying influencing factors, the findings have been diverse. Few studies have developed prediction models for DOL risk in this population.

In this study, we incorporate a wider range of demographic variables while specifically accounting for the clinical characteristics of GDM. Our primary objective is to construct a predictive model for the risk of DOL among mothers with GDM. Using the model for the early identification of high-risk individuals aims to facilitate timely interventions to prevent DOL. The ultimate goal is to assist healthcare professionals in implementing targeted preventive strategies, thereby reducing the incidence of DOL and promoting successful breastfeeding.

## 2 Study sample and methods

### 2.1 Study participants and sample size estimation

This was a prospective study. Mothers with GDM who were hospitalized in the obstetrics departments of seven tertiary (grade 3A) hospitals across five cities in Guangdong Province, China, between October 10, 2023, and December 8, 2024, were selected using convenience sampling.

The criteria for inclusion were as follows: (1) pregnant women diagnosed with GDM; (2) women with the intention of breastfeeding;(3)age ≥20 years;(4)possessing basic literacy skills with normal cognition.

Exclusion criteria were as follows: (1) mother with contraindications to breastfeeding; (2) mother-infant separation; (3) mother with any serious perinatal complications such as hypertensive disorders; (4) psychiatric disorders; (5) mother with incomplete clinical information.

Sample size estimation: The sample size was calculated with reference to the logistic regression formula *N*= (*n*×10)/*I* (15). Assuming n=7 candidate predictors and an anticipated DOL incidence (*I*) of 25.2%, the initial estimate yielded 278 participants. To accommodate a 7:3 training-validation split and a 10% potential attrition rate, the target sample size was increased to 443 (training: 310; validation: 133). In total, 511 participants were enrolled.

This study was approved by the Ethics Committee of the First Affiliated Hospital of Sun Yat-sen University (approval no. [2023]621).

### 2.2 Study methods

#### 2.2.1 Study tools

##### 2.2.1.1 General data

Data were collected using structured questionnaires and extracted from electronic medical records. The collected variables are summarized below: (1) Baseline Maternal Characteristics: age, marital status, education level, occupation, residential location, and household income, pre-pregnancy body mass index (BMI), smoking status, blood lipid profile, and experience of stressful life events during pregnancy. (2) Pregnancy and Delivery Variables: parity, gestational weeks at delivery, GDM treatment regimen, and glycemic control status, mode of delivery and use of labor analgesia. (3) Neonatal Outcomes and Postpartum Breastfeeding Practices: sex, birth weight, and blood glucose level, maternal breastfeeding experience, receipt of breastfeeding knowledge training, intended feeding method, rooming-in practice, timing of first suckling, breastfeeding frequency within the first 24 hours and between 24~48 hours postpartum, and use of formula supplementation prior to lactogenesis initiation.

##### 2.2.1.2 Breastfeeding assessment

The LATCH Scale is a widely used tool for breastfeeding assessment (16). Its name derives from the first letters of five key components: Latch (L), Audible swallowing (A), Nipple Type (T), maternal Comfort (C), and Help needed (H). Each component is scored on a scale from 0 to 2, with the total scores ranging from 0~10; where a higher score describes better breastfeeding proficiency. The scale demonstrates high reliability, with Cronbach’s α coefficients ranging from 0.87 to 0.95 (17).

##### 2.2.1.3 Screening for depression

Depressive symptoms were assessed using the Edinburgh Postnatal Depression Scale (EPDS) (18), which is the most widely used screening tool for postpartum depression. The EPDS comprises 10 items, which can be grouped into three dimensions: emotional deficiency, anxiety, and depression. Each item is scored from 0 to 3, yielding a total score ranging from 0 to 30; higher scores indicate more severe depressive symptoms. The scale has demonstrated good reliability in the present study, with a Cronbach’s α coefficient of 0.86.

##### 2.2.1.4 Screening for anxiety

The maternal anxiety status was evaluated using Zung’s Self-Rating Anxiety Scale (SAS) (19). The SAS comprises 20 items rated on a 1~4 point scale (including 5 reverse-scored items). The raw total score is obtained by summing all item scores, which is then multiplied by 1.25 and rounded to the nearest integer to yield the standard score. The SAS demonstrates acceptable reliability with a Cronbach’s α coefficient of 0.733.

##### 2.2.1.5 Social support level

The maternal social support status was evaluated using the Social Support Rating Scale (SSRS) developed by Shui-yuan Xiao et al. (20). The SSRS consists of 10 items that comprehensively evaluate an individual’s social support level across three dimensions: objective support, subjective support and utilization of social support. With a total possible score of 66 points, the SSRS demonstrates a positive correlation between score and social support level. The scale shows excellent reliability with a Cronbach’s α coefficient of 0.92.

##### 2.2.1.6 Sleep quality

Sleep quality was assessed using the Pittsburgh Sleep Quality Index (PSQI) (21). Developed by Buysse et al., this 7-component instrument measures the following domains: subjective sleep quality, sleep latency, sleep duration, sleep efficiency, sleep disturbances, use of sleeping medications, and daytime dysfunction. Each component is scored from 0 to 3, yielding a global score ranging from 0 to 21, with higher scores indicating poorer sleep quality. In the present study, the Chinese version of the PSQI demonstrated good reliability, with a Cronbach’s α coefficient of 0.842. Its diagnostic utility is supported by a reported sensitivity of 98.3% and specificity of 90.2%.

#### 2.2.2 Data collection and quality control methods

Prior to the survey, the principal investigator provided detailed explanations to the leaders of each sub-center who were trained on filling out the questionnaires and risk evaluation so as to ensure consistency across the study. During data collection, the investigators followed the criteria for inclusion and exclusion when selecting participants for the study, explaining the objectives and procedures involved in the study. The leaders of each sub-center regularly reported research progress. After data collection, these leaders reviewed the quality of questionnaire and promptly supplemented any missing items. The study used standardized instructions for on-site questionnaire distribution, with immediate checks for completeness to ensure data quality. On the first day postpartum, we conducted assessments of breastfeeding, depression symptoms, anxiety status, social support level, and sleep quality, and collected the most recent pre-delivery routine laboratory test results (i.e., from the final prenatal examination).

### 2.3 Statistical analysis

The data were processed and analyzed using SPSS (version 26.0) and R (version 4.4.1) software. Continuous variables with a normal distribution are presented as mean ± standard deviation (mean ± SD) and were compared using the Student’s t-test. Continuous variables with a non-normal distribution are expressed as median and interquartile range [M (IQR)] and were compared using the Mann-Whitney U test. Categorical variables are described as frequency and percentage (n, %), and inter-group comparisons were performed using the Chi-square test or Fisher’s exact test, as appropriate.

In univariate analysis, independent variables with statistical significance (p < 0.1) were included in a least absolute shrinkage and operator selection(LASSO) regression analysis and logistic regression analysis. The variance inflation factor (VIF) was used in collinearity diagnosis to assess multi-collinearity, with VIF > 10 indicating collinearity. A predictive equation was then constructed based on the partial regression coefficients of various variables, and the prediction model for DOL risk of mothers with GDM was created. R (version 4.4.1) software was used to construct the nomogram, and internal validation of the model was done using the Bootstrap self-sampling method.

The Hosmer-Lemeshow (H-L) test and calibration curves were used to assess model calibration. Receiver operator characteristic (ROC) curves were plotted, and the area under the curve (AUC) was calculated along with sensitivity and specificity to evaluate discriminative performance. Clinical decision curve analysis (DCA) was conducted to evaluate the model’s clinical utility by calculating net benefits at different threshold probabilities.

## 3 Results

### 3.1 The incidence of delayed onset of lactogenesis among mothers with gestational diabetes mellitus

This study ultimately included 511 mothers with GDM, of whom 136 experienced DOL, yielding an incidence of 26.61%.

### 3.2 Univariate analysis of DOL risk factors among mothers with gestational diabetes mellitus

The 344 mothers in the training cohort were stratified into non-DOL and DOL groups for univariate analysis. The analysis revealed significant differences (P < 0.05) between the two groups in the following nine variables: pre-delivery BMI, EPDS score, SAS score, PSQI score, SSRS score, serum albumin levels, primary feeding method within 48 hours postpartum, LATCH score, and blood glucose control level during pregnancy. These variables may constitute risk factors for DOL among mothers with GDM. The detailed results are presented in **Supplementary Table 1**.

### 3.3 LASSO regression analysis of delayed onset of lactogenesis among women with gestational diabetes mellitus

**Figure 1:**
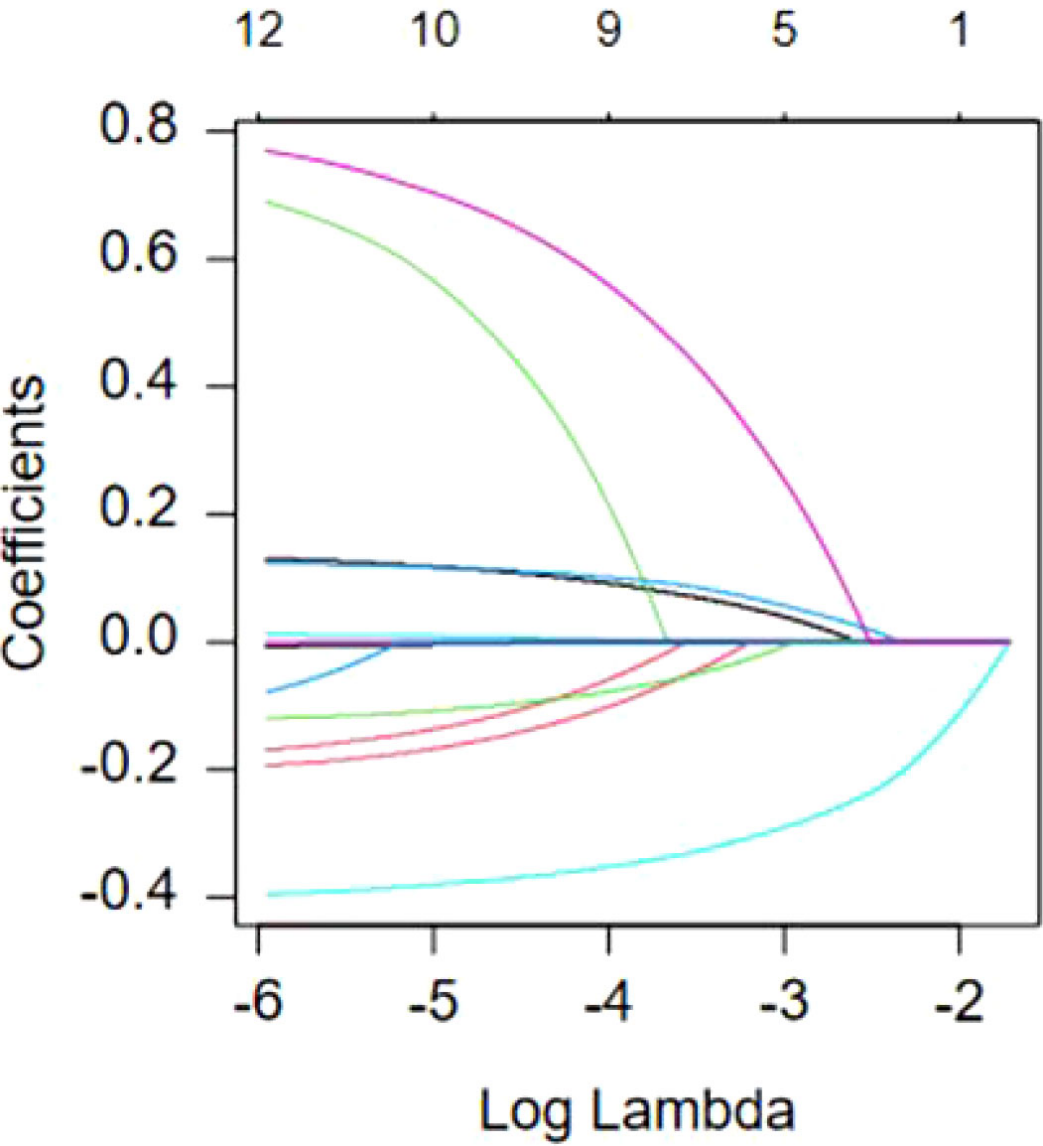
Coefficient variation trends of variable selection in LASSO regression.

**Figure 2:**
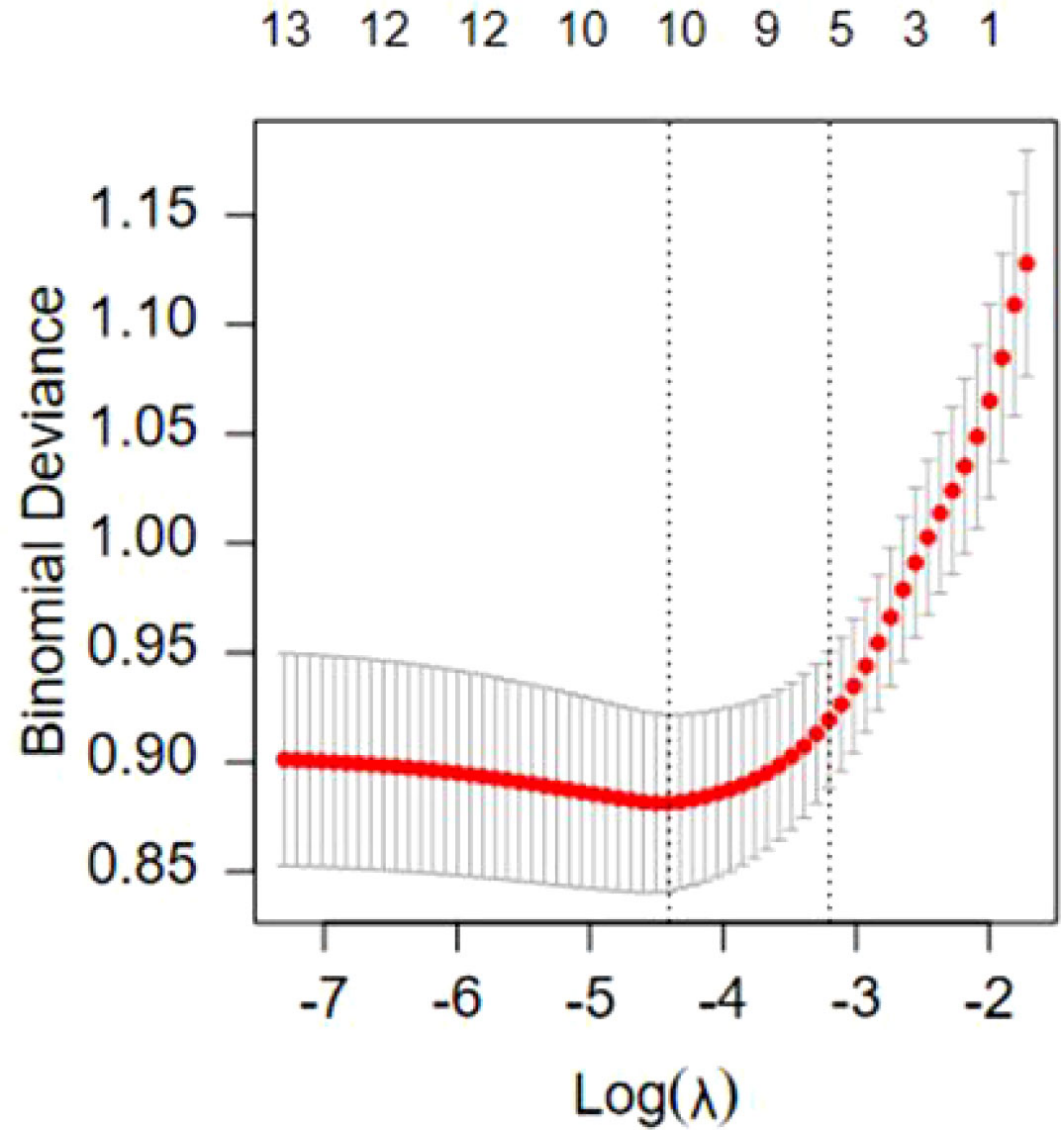
LASSO regression cross-validation curve.

This study employed LASSO regression analysis to further identify predictive variables. Variables with *P* value<0.1 in the univariate analysis were included in the LASSO regression. Through 10-fold cross-validation, optimal predictive variables were selected for the model. In this study, ten non-zero coefficients were screened using Lambda.min as the criterion, including pre-delivery BMI, breastfeeding frequency within 24-48 hours, rooming-in, EPDS score, SAS score, SSRS score, the results of 2h OGTT, serum albumin levels, LATCH score, and blood glucose control during pregnancy. **Figures 1**, **2** for details.

### 3.4 Logistic regression analysis of DOL among mothers with GDM

Using the occurrence of DOL as a binary dependent variable, the 10 variables selected by LASSO regression were analyzed through logistic regression. Five variables that had a *p* value <0.05, including pre-delivery BMI, EPDS score, serum albumin levels, LATCH score, blood glucose control during pregnancy were ultimately identified as significant risk factors for DOL among mothers with GDM. The results are presented in **Table 1**.

**Table 1:** Logistic regression analysis of DOL among mothers with GDM.

Intercept and variate	*β*	*SE*	*z*	*OR*(95%*CI*)	*P*- value
Intercept	1.163	2.447	0.475		0.634
Pre-delivery BMI	0.135	0.044	3.061	1.145 (1.052,1.252)	0.002
EPDS score	0.147	0.040	3.697	1.159 (1.073,1.255)	<0.001
Serum albue levels	-0.131	0.060	-2.175	0.877 (0.779,0.987)	0.030
LATCH score	-0.420	0.072	-5.854	0.657 (0.569,0.754)	<0.001
Blood glucose control during pregnancy					
good				reference	
poor	0.802	0.301	2.660	2.230 (1.239,4.056)	0.008

The variance inflation factor (VIF) was used to diagnose multicollinearity, with VIF > 10 indicating collinearity. All VIF values were below 10, indicating no significant multicollinearity existed between variables.

### 3.5 Construction of a prediction model for delayed onset of lactogenesis among mothers with gestational diabetes mellitus

The following logistic regression equation was constructed based on the identified risk factors: Logit(*P*)= 1.163 + 0.138 ×X_(pre-delivery BMI )_+ 0.148×X _(EPDS score)_ - 0.132×X _(serum albumin levels)_ - 0.413×X _(LATCH score)_ + 0.773×X _(blood glucose level during pregnancy)_.

**Figure 3:**
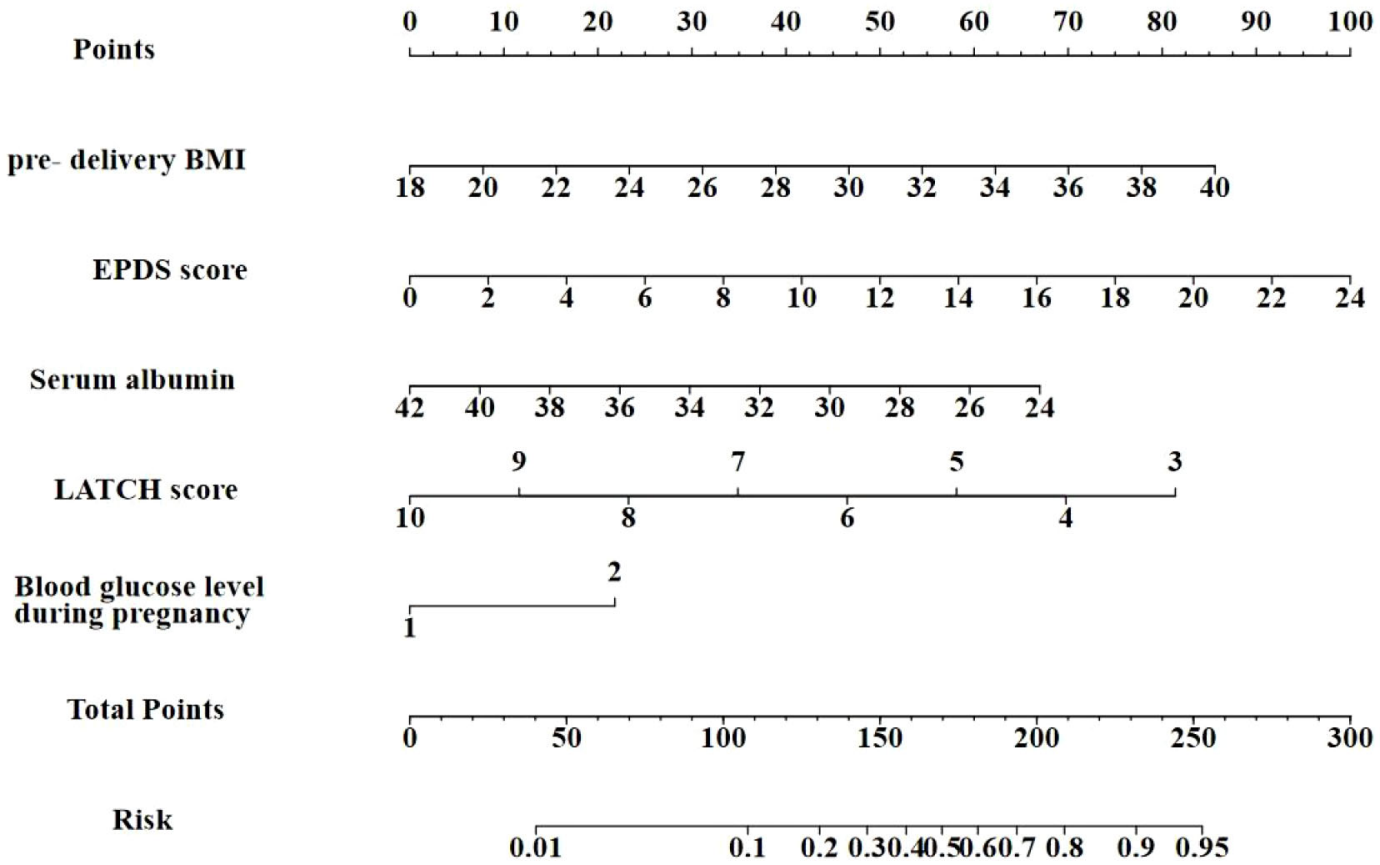
Nomogram of DOL risk factors among mothers with GDM.

Using R (version 4.4.1) software, a nomogram was created to visually represent the prediction model (**Figure 3**). The regression coefficients for each factor were used to assign scores. Each variable was aligned with a vertical score scale, allowing for individual factor scores to be determined. The total score, which was the sum of the individual scores, was then converted into DOL probability through a functional relationship. The corresponding score (top line) is found according to the value of each predictor variable (the line after each variable), then the values of the individual scores are summed to obtain the total score, and the corresponding predicted probability is based on the total score (bottom line).

### 3.6 Evaluation and verification of the prediction model for delayed onset of lactogenesis risk among mothers with GDM

**Figure 4:**
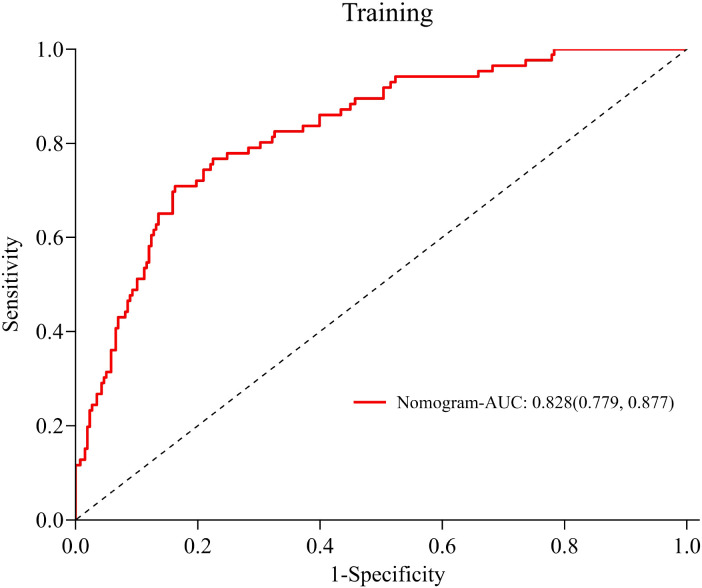
ROC curve of the DOL risk prediction model for mothers with GDM.

**Figure 5:**
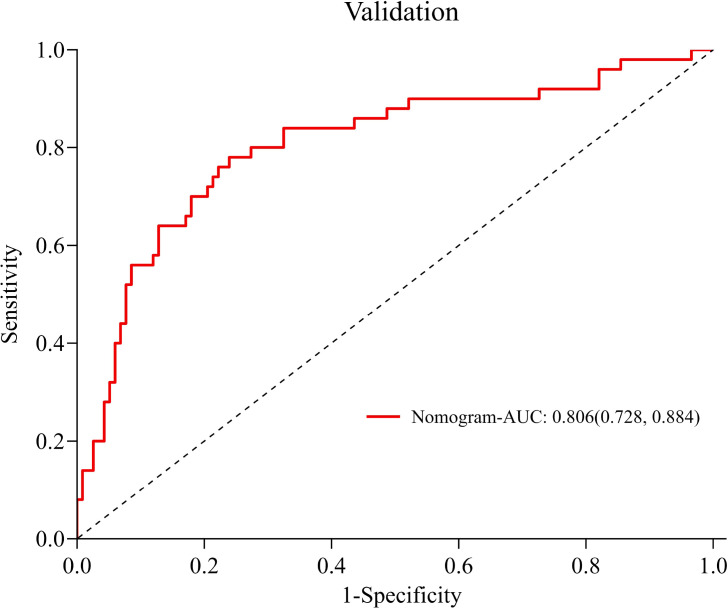
Internal validation of DOL risk prediction model for mothers with GDM.

**Figure 6:**
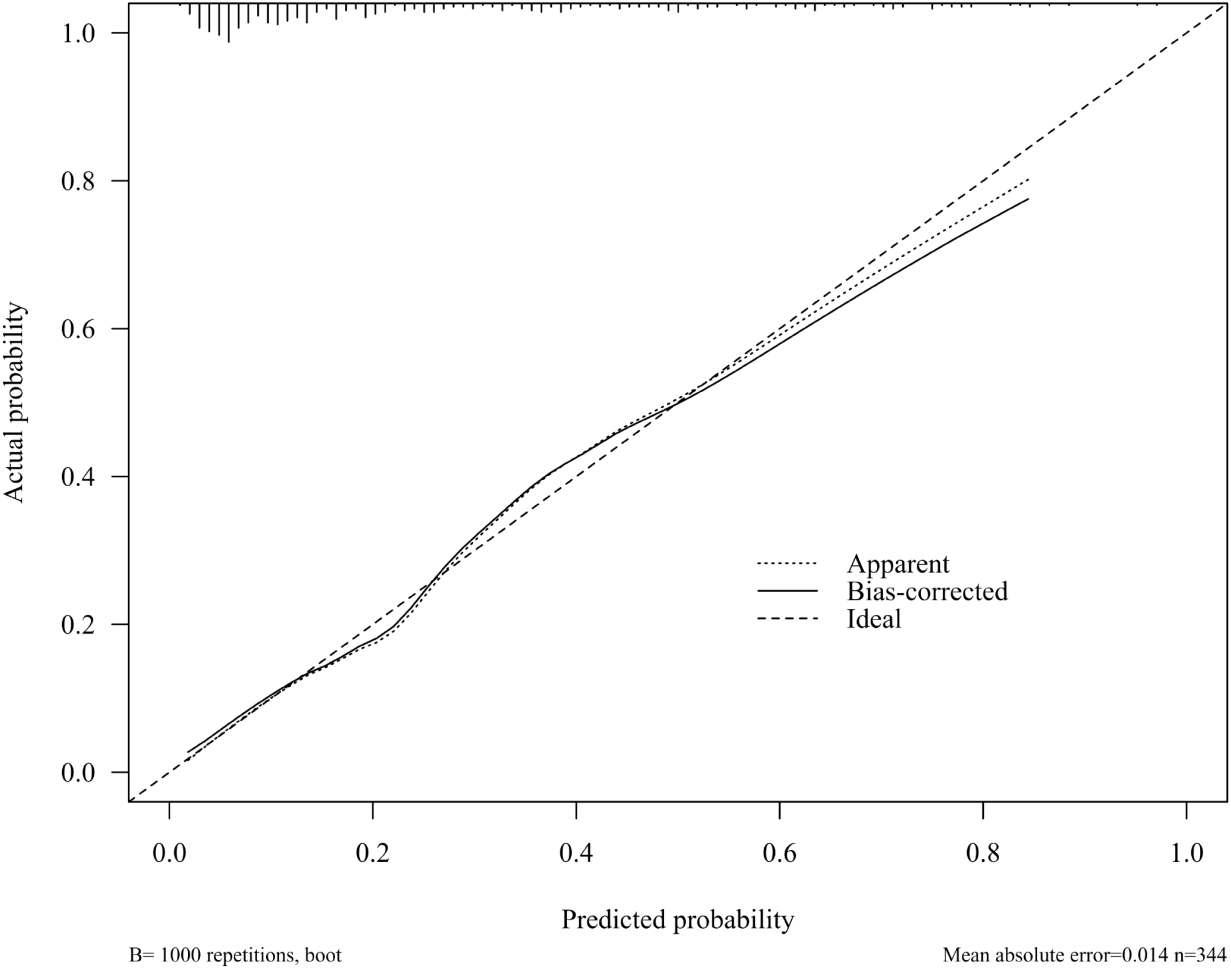
Calibration curve diagram of DOL risk prediction model for mothers with GDM.

**Figure 7:**
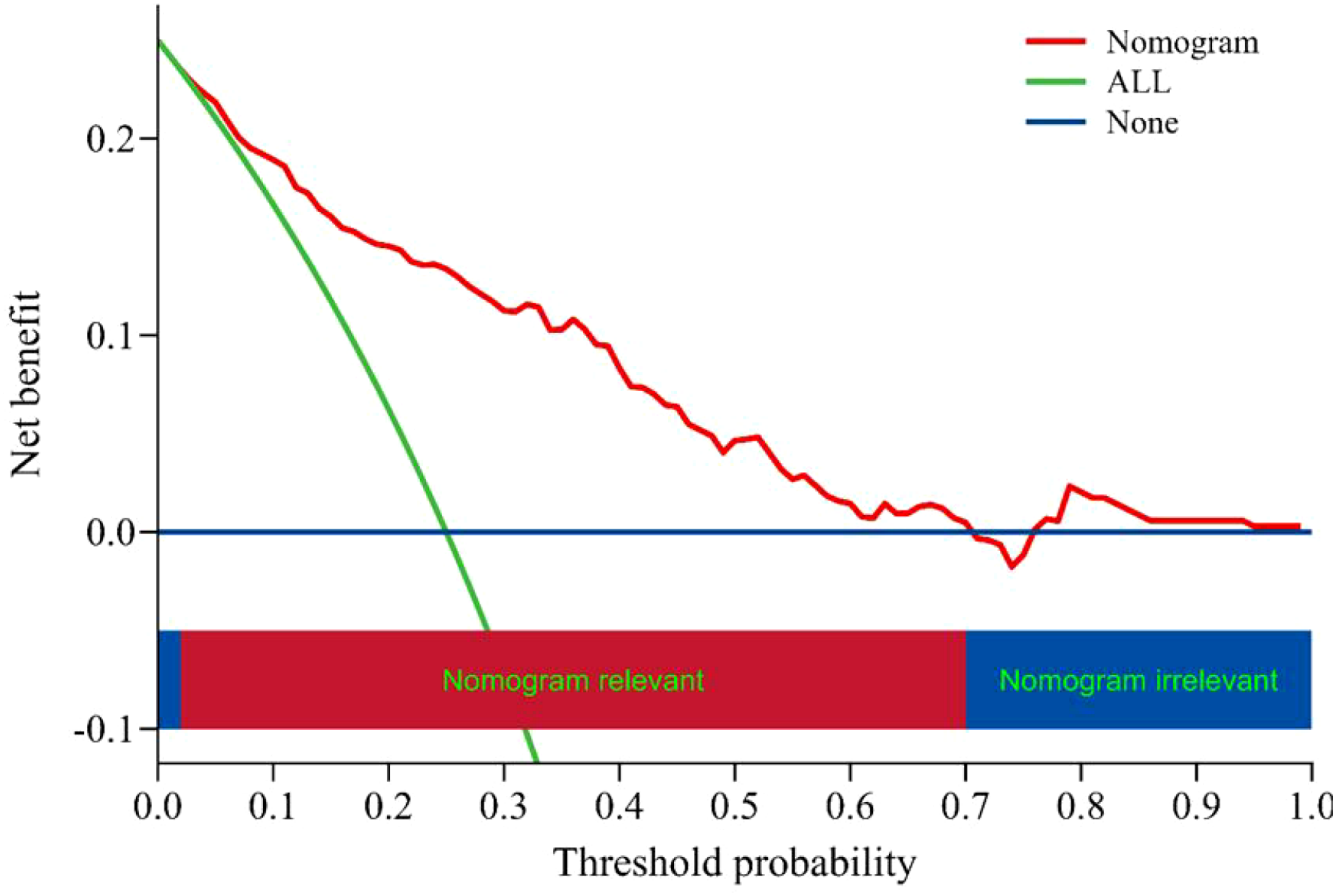
Clinical decision curve analysis of DOL risk prediction model for mothers with GDM.

The ROC curve was drawn (**Figure 4**). The AUC was 0.828 (95% *CI*:0.779-0.877), indicating good predictive capacity. At the optimal cut-off point of 0.341, the model demonstrated a sensitivity of 70.9%, a specificity of 83.7%, and a maximum Youden index of 0.546. The internal validation of the model yielded an AUC of 0.806 (95% CI: 0.728-0.884) (**Figure 5**), with a sensitivity of 78.0% and a specificity of 76.1%. The Hosmer-Lemeshow goodness-of-fit test showed χ2 = 7.226, *P* = 0.546, indicating good calibration. The calibration curve diagram (**Figure 6**) showed that the calibration and standard curves were almost overlapping, indicating that the predicted DOL risk probabilities closely matched the observed probabilities. The DCA (**Figure 7**) showed that the clinical decision curve was consistently above the invalid lines, indicating the model’s potential clinical effectiveness when using interventions for individuals who have a DOL risk probability in the range of 0.03 to 0.71.

## 4 Discussion

### 4.1 Analysis of DOL occurrences among mothers with GDM

This study included a total of 511 parturients, among whom 167 experienced DOL, yielding an overall incidence of 26.61%. This rate was somewhat higher than that reported in the study by Luo Fengjuan et al. (14). Notably, Luo’s study population was recruited from secondary (grade 2A) hospitals, whereas our subjects were enrolled from seven tertiary (grade 3A) hospitals in Guangdong Province. Tertiary (grade 3A) hospitals typically manage cases with more comorbidities and complex conditions, which may increase breastfeeding difficulties and consequently reduce maternal motivation and willingness to breastfeed. However, our findings were lower than the 34.0% incidence reported in the systematic review (22). This discrepancy may be attributed to differences in the study populations. The research subjects of the latter come from diverse regions (United States, Brazil, India and China), where variations in dietary habits, cultural perspectives on childbirth, and breastfeeding practices could significantly influence lactation outcomes.

### 4.2 Analysis of DOL risk factors among mothers with GDM

#### 4.2.1 mothers with GDM who were poor glycemic control during pregnancy were at greater risk of DOL

The results of this study showed that mothers with GDM who were Poor glycemic control during pregnancy were at greater risk of DOL. During the second and third trimesters, GDM women develop various insulin-resistant substances that reduce bioactive insulin levels. This insulin insufficiency decreases the insulin-to-glucose (I/G) ratio, thereby diminishing mammary gland insulin utilization and inhibiting α-lactalbumin transcription and lactose synthesis (23). As lactose serves as a critical factor for lactogenesis initiation, its reduced synthesis delays the onset of lactation. Furthermore, insulin exerts positive effects on prolactin production by promoting prolactin mRNA accumulation and gene transcription (24). Thus, relative insulin deficiency compromises lactation establishment. We hypothesize that the severity of insulin deficiency in mothers with poorly controlled glucose is directly proportional to the probability of developing DOL. We hypothesize that the degree of insulin deficiency is more pronounced in pregnant women with suboptimal glycemic control. Additionally, poor glycemic control during pregnancy increases the risks of adverse perinatal outcomes including preterm birth, placental abruption, preeclampsia, neonatal hypoglycemia, birth asphyxia, and macrosomia (25, 26), all of which may potentially delay lactogenesis initiation. Consequently, gravidas should perform regular self-monitoring of blood glucose, engage in appropriate physical activity, and maintain a diabetes-specific diet to achieve optimal glycemic control, thereby reducing the risk of DOL.

#### 4.2.2 Elevated LATCH scores and serum albumin levels could significantly decrease DOL risk among mothers with GDM

Our findings in this study revealed that LATCH scores among mothers in the Non-DOL group were significantly higher than those in the DOL group, suggesting that higher LATCH scores are associated with a lower DOL risk. The seminal work by Krishna et al. (27) showed that LATCH scores serves as a robust metric for assessing breastfeeding, with higher scores demonstrating a significant association with increased exclusive breastfeeding rates. The reason for this may be that mothers with higher LATCH scores have greater breastfeeding self-efficacy, more confidence in facing challenges, and a stronger motivation to breastfeed. This heightened willingness promotes milk production. The LATCH scale can comprehensively and systematically assess maternal breastfeeding performance within a short time frame. By providing an objective quantitative evaluation, it helps specify breastfeeding problems and effectively identifies individuals requiring breastfeeding support. Therefore, it is recommended to use the LATCH scale to assess mothers’ breastfeeding proficiency during the early postpartum period. For mothers with suboptimal LATCH scores, early implementation of lactogenesis initiation strategies is strongly recommended.

Additionally, we found that higher serum albumin levels correlated with lower DOL risk among mothers with GDM. Previous research has demonstrated the association between serum albumin levels and DOL risk, wherein lower levels correlate with higher risk. Hypoalbuminemia constitutes a significant risk factor for DOL (28). Serum albumin, a long-term biomarker of nutritional status, reflects maternal nutrient reserves. During pregnancy, adequate nutritional intake is crucial for supporting fetal-maternal development and promoting mammary gland growth and maturation, which in turn helps ensure postpartum lactation capacity and milk quality. This result aligns with international research. Consequently, regular monitoring of serum albumin levels coupled with increased intake of high-quality protein is recommended to maintain optimal albumin concentrations, thereby reducing the incidence of DOL.

#### 4.2.3 Higher pre-delivery BMI and elevated EPDS scores were associated with an increased risk of DOL among mothers with GDM

Our findings in this study revealed that pre-delivery BMI among mothers in the DOL group was significantly higher than that in the Non-DOL group, demonstrating that higher pre-delivery BMI is associated with greater DOL risk. Given that gestational weight gain impacts both short- and long-term maternal and neonatal health outcomes, greater emphasis should be placed on weight management during pregnancy (29). The underlying mechanisms may involve two key aspects. Firstly, progesterone stored in adipose tissue interferes with prolactin receptor function in mammary alveolar cells, and women with higher BMI maintain more higher progesterone levels, potentially prolonging the inhibition of lactogenesis (30). Secondly, excessive adipose tissue in the breasts of obese women may impede milk flow through the ducts, while their body morphology often makes proper breastfeeding positioning more challenging. These combined difficulties decrease the likelihood of sustaining breastfeeding (30). Therefore, Increased attention should be devoted to gestational weight management, including regular prenatal check-ups for weight monitoring and intensified health education, to prevent excessive gestational weight gain.

We also found that mothers with higher EPDS scores were at higher risk of DOL. As the most common perinatal psychological disorder, postpartum depression not only compromises maternal physical and mental health but also adversely affects breastfeeding practices. This relationship may be explained through two mechanisms. Firstly, research indicates that mothers with depression often experience maternal role maladjustment, and their negative emotions can undermine breastfeeding confidence, thereby increasing the risk of DOL. This aligns with findings confirming an inverse relationship between depression scores and breastfeeding self-efficacy (31). Secondly, lactogenesis is regulated by multiple hormones including prolactin, oxytocin, and cortisol. Prolactin constitutes the principal hormonal regulator of mammary gland lactogenic function, playing an indispensable role in both the initiation and maintenance of lactogenesis. Its absence results in the complete failure of milk production (32). Oxytocin triggers the let-down reflex by contracting myoepithelial cells surrounding alveoli, which is crucial for milk ejection. Cortisol, the primary glucocorticoid in humans, synergizes with prolactin to initiate nuclear transcription and milk protein synthesis (33). Maternal depression disrupts the secretion of these lactation-related hormones, thereby impairing both milk synthesis and lactogenesis initiation. Currently, China lacks specialized emotional counseling clinics for postpartum mothers, which reflects insufficient societal attention to perinatal psychological well-being. It is recommended to implement systematic screening for postpartum depression and initiate early intervention strategies for high-risk populations.

### 4.3 The prediction model of DOL risk among mothers with GDM had relatively good predictive performance

Given the numerous DOL risk factors among mothers with GDM and the associated high incidence of DOL, we used logistic regression in this study to analyze DOL risk factors and constructed a nomogram. This model was found to perform well in terms of its predictive ability and clinical effectiveness, with a relatively high AUC of 0.828 (95% *CI*: 0.779-0.877).

Following internal validation, the model achieved an AUC of 0.806 (95% *CI*: 0.728-0.884), reflecting a good discrimination. The prediction model demonstrated a sensitivity of 70.9%, showing that it had a strong ability to correctly identify mothers with GDM. This feature is especially advantageous for clinical settings, as it allows healthcare providers to identify high-risk individuals more accurately, facilitating more targeted lactation support strategies.

The goodness of fit of the model was confirmed by the Hosmer-Lemeshow test, which yielded a *χ*^2^ of 7.226 and a *p* value of 0.546, indicating a good agreement between the model’s predicted DOL risk and actual outcomes. Additionally, the calibration curve closely followed the reference line, indicating that the model’s predicted DOL rates closely aligned with the actual observed DOL rates, demonstrating its relatively good calibration ability.

### 4.4 Clinical implications of a prediction model for DOL in women with GDM

In existing research, Luo Fengjuan et al. (14) developed a prediction model for DOL in GDM patients. However, this model has limitations including a restricted set of variables, weak association with GDM pathophysiology, and a single-center study population, which constrain its generalizability and require further validation. In contrast, this study established a multi-center cohort from seven tertiary hospitals in Guangdong Province, systematically integrated multi-dimensional clinical factors, and constructed a novel prediction model for DOL risk in GDM patients. Validation results demonstrate that the model exhibits excellent predictive performance and clinical applicability, providing healthcare professionals with a reliable risk assessment tool.

The results of this study indicate that the optimal cut-off value for the prediction model was 0.341, corresponding to a score of 151 points. Based on this threshold, subjects can be categorized into two groups: those with scores below 151 points are classified as low-risk for DOL, while those with scores above 151 points are classified as high-risk for DOL. It is recommended that clinicians implement differentiated interventions according to this risk stratification. For high-risk mothers at risk of DOL, systematic lactation initiation strategies should be initiated as early as possible. It is recommended to establish standardized referral and handover procedures to seamlessly integrate this high-risk population into the community healthcare service system, ensuring they receive continuous professional support, thereby minimizing the incidence of delayed onset of lactation (34).

## 5 Conclusion

This study analyzed data from GDM patients who delivered at seven tertiary (grade 3 A)hospitals in Guangdong Province in China. The results revealed a relatively high incidence of DOL among GDM mothers. Higher pre-delivery BMI and EPDS score were associated with increased DOL risk, while elevated serum albumin levels and LATCH score were protective factors. Poor glycemic control during pregnancy was also identified as a significant risk factor for DOL. The developed risk prediction model for DOL in GDM mothers demonstrated excellent calibration and discrimination. A nomogram was created to visually present the prediction results, enabling healthcare providers to quickly and efficiently identify high-risk individuals. This tool provides a theoretical foundation for developing clinical strategies to promote timely lactogenesis.

However, this study had the following limitations: (1) The use of convenience sampling may introduce selection bias, as it tends to include pregnant women with regular prenatal visits and higher compliance, which could affect the accurate estimation of certain predictive factor effects; (2) Although the model was developed using multi-center data, external validation has not yet been conducted. Consequently, the favorable discrimination and calibration performance observed in the current development set may not be generalizable to new populations with distinct demographic characteristics. Future research should further validate the model’s generalizability and clinical applicability by incorporating international multi-center data, along with conducting more extensive and rigorous external validation studies.

## Data Availability

The original contributions presented in the study are included in the article/**Supplementary Material**. Further inquiries can be directed to the corresponding author.

## References

[1] Sweeting A Wong J Murphy HR Ross GP . A clinical update on gestational diabetes mellitus. Endocr Rev. (2022) 43:763–793. doi: 10.1210/endrev/bnac003, PMID: 35041752 PMC9512153

[2] Kim KS Hong S Han K Park CY . The clinical characteristics of gestational diabetes mellitus in Korea: A national health information database study. Endocrinol Metab (Seoul). (2021) 36:628–636. doi: 10.3803/EnM.2020.948, PMID: 34034366 PMC8258326

[3] Wu YX Wang ZL . Hyperglycemia in pregnancy: diagnosis, monitoring and prevention. Chin J Gen Practitioners. (2022) 21:624–629. doi: 10.3760/cma.j.cn114798-20220330-00246, PMID: 40668938

[4] Magliano DJ Boyko EJDA . IDF Diabetes Atlas. Brussels: International Diabetes Federation (2021).

[5] Masi AC Stewart CJ . Role of breastfeeding in disease prevention. Microb Biotechnol. (2024) 17:e14520. doi: 10.1111/1751-7915.14520, PMID: 38946112 PMC11214977

[6] Victora CG Bahl R Barros AJD França GVA Horton S Krasevec J . Breastfeeding in the 21st century: epidemiology, mechanisms, and lifelong effect. Lancet. (2016) 387:475–490. doi: 10.1016/S0140-6736(15)01024-7, PMID: 26869575

[7] Poston L Caleyachetty R Cnattingius S Corvalán C Uauy R Herring S . Preconceptional and maternal obesity: epidemiology and health consequences. Lancet Diabetes Endocrinol. (2016) 4:1025–1036. doi: 10.1016/S2213-8587(16)30217-0, PMID: 27743975

[8] World Health Organization . (2017). Guideline: Protecting, Promoting and Supporting Breastfeeding in Facilities Providing Maternity and Newborn Services. Geneva: World Health Organization. 29565522

[9] World Health Organization . (2023). Infant and young child feeding. Available online at: https://www.who.int/news-room/fact-sheets/detail/infant-and-young-child-feeding (Accessed March 20, 2026).

[10] Rocha BO Machado MP Bastos LL Barbosa Silva L Santos AP Santos LC . Risk factors for delayed onset of lactogenesis II among primiparous mothers from a Brazilian baby-friendly hospital. J Hum Lact. (2020) 36:146–156. doi: 10.1177/0890334419835174, PMID: 30901295

[11] Zhan YJ Zhou MF . Research progress on delayed onset of lactogenesis in gestational diabetes mellitus women. Chin J Health Manage. (2024) 19:225–229. doi: 10.3760/cma.j.cn115624-20231023-00217, PMID: 40668938

[12] Li S Wupuer T Hou R . Factors influencing delayed onset of lactogenesis: A scoping review. Int J Gen Med. (2024) 17:2311–26. doi: 10.2147/IJGM.S452108, PMID: 38799202 PMC11127660

[13] Wu JL Pang SQ Jiang XM Zheng QX Han XQ Zhang XY . Gestational diabetes mellitus and risk of delayed onset of lactogenesis: A systematic review and meta-analysis. Breastfeed Med. (2021) 16:385–392. doi: 10.1089/bfm.2020.0356, PMID: 33891507

[14] Luo FJ Bao NZ Li SH . Establishment and validation of a predictive model for therisk of delayed lactation initiation in pregnant women withgestational diabetes mellitus. J Clin Pathological Res. (2020) 40:1394–400.

[15] Peduzzi P Concato J Kemper E Holford TR Feinstein AR . A simulation study of the number of events per variable in logistic regression analysis. J Clin Epidemiol. (1996) 49:1373–1379. doi: 10.1016/S0895-4356(96)00236-3, PMID: 8970487

[16] Sowjanya S Venugopalan L . LATCH score as a predictor of exclusive breastfeeding at 6 weeks postpartum: A prospective cohort study. Breastfeed Med. (2018) 13:444–9. doi: 10.1089/bfm.2017.0142, PMID: 29957025

[17] Gerçek E Sarıkaya Karabudak S Ardıç Çelik N Saruhan A . The relationship between breastfeeding self-efficacy and LATCH scores and affecting factors. J Clin Nurs. (2017) 26:994–1004. doi: 10.1111/jocn.13423, PMID: 27272098

[18] Cox JL Holden JM Sagovsky R . Detection of postnatal depression. Development of the 10-item Edinburgh Postnatal Depression Scale. Br J Psychiatry. (1987) 150:782–6. doi: 10.1192/bjp.150.6.782, PMID: 3651732

[19] Zung WW . A rating instrument for anxiety disorders. Psychosomatics. (1971) 12:371–9. doi: 10.1016/S0033-3182(71)71479-0, PMID: 5172928

[20] Xiao SY . Theoretical basis and research applications of the social support rating scale. J Clin Psychiatry. (1994), 98–100.

[21] Buysse DJ Reynolds CF III Monk TH Berman SR Kupfer DJ . The Pittsburgh Sleep Quality Index: a new instrument for psychiatric practice and research. Psychiatry Res. (1989) 28:193–213. doi: 10.1016/0165-1781(89)90047-4, PMID: 2748771

[22] Li YS Hu SS Liu J . A systematic review of the incidence and influencing factors of delayed onset of lactogenesis among women with gestational diabetes mellitus. J Mudanjiang Med Univ. (2021) 42:40–46. doi: 10.13799/j.cnki.mdjyxyxb.2021.06.009

[23] Nommsen-Rivers LA Dolan LM Huang B . Timing of stage II lactogenesis is predicted by antenatal metabolic health in a cohort of primiparas. Breastfeed Med. (2012) 7:43–9. doi: 10.1089/bfm.2011.0007, PMID: 21524193 PMC3546359

[24] Mahajan MA Stanley FM . Insulin-activated Elk-1 recruits the TIP60/NuA4 complex to increase prolactin gene transcription. Mol Cell Endocrinol. (2014) 382:159–69. doi: 10.1016/j.mce.2013.09.021, PMID: 24075908

[25] Liu B Cai J Xu Y Long Y Deng L Lin S . Early diagnosed gestational diabetes mellitus is associated with adverse pregnancy outcomes: A prospective cohort study. J Clin Endocrinol Metab. (2020) 105. doi: 10.1210/clinem/dgaa633, PMID: 32898218

[26] Wang ZL Chen HT . Poor control of blood glucose and adverse pregnancy outcome. China J Obstetrics Gynecology. (2020) 36:405–408. doi: 10.19538/j.fk2020050106

[27] Krishna PV Devi LAVV Silpa G Teja R Bhupathi S . A study on the role of LATCH score as a predictor of exclusive breastfeeding at 6 weeks and 10 weeks in postpartum primigravida in a tertiary care centre. J Cardiovasc Dis Res. (2023) 14:955–961.

[28] Lian W Ding J Xiong T Liuding J Nie L . Determinants of delayed onset of lactogenesis II among women who delivered via Cesarean section at a tertiary hospital in China: a prospective cohort study. Int Breastfeed J. (2022) 17:81. doi: 10.1186/s13006-022-00523-3, PMID: 36451171 PMC9714018

[29] Lasserre-Laso N Leiva-Manzor G Bustos-Arriagada E Etchegaray-Armijo K . Association between exclusive breastfeeding, nutritional status and eating behavior, in Chilean schoolchildren: A cross-sectional study. Nutrients. (2025) 17. doi: 10.3390/nu17213444, PMID: 41228513 PMC12609394

[30] Zhang F Dong L Zhang CP Li B Wen J Gao W . Increasing prevalence of gestational diabetes mellitus in Chinese women from 1999 to 2008. Diabetes Med. (2011) 28:652–657. doi: 10.1111/j.1464-5491.2010.03205.x, PMID: 21569085

[31] Li L Xiao G Peng HT Zhang TT Zhou MJ Qin CX . Research progress on the impacts of depression in different stages of perinatal period on exclusive breastfeeding. Chin Nurs Manage. (2021) 21:1871–1875. doi: 10.3969/j.issn.1672-1756.2021.12.021, PMID: 35900448

[32] Zhang MH Gao XL Sun Y . Breastfeeding and Human Lactation. Beijing: People’s Medical Publishing House (2021).

[33] Thul TA Corwin EJ Carlson NS Brennan PA Young LJ . Oxytocin and postpartum depression: A systematic review. Psychoneuroendocrinology. (2020) 120, 104793. doi: 10.1016/j.psyneuen.2020.104793, PMID: 32683141 PMC7526479

[34] Hurley SM Whyte K Sorensen J . Cost-Effectiveness and Equity Trade-Off for Breastfeeding Interventions. London, UK: IntechOpen (2024). doi: 10.5772/intechopen.110715, PMID:

