# Supplementary material for: Development and validation of a risk prediction model of delayed onset of lactogenesis among mothers with gestational diabetes mellitus: a prospective multicenter study
Source: Front Clin Diabetes Healthc. 2026 Mar 26;7:1721649. doi: 10.3389/fcdhc.2026.1721649 (PMC13061669; doi:10.3389/fcdhc.2026.1721649)
Supplement: Supplementary file 1 [file DataSheet1.doc]

Supplementary TABLE 1 Univariate analysis of DOL among mothers with GDM

| **Rike factor** | **Non-DOL**  **(*n* = 258)** | **DOL**  **(*n* = 86)** | **Statistic** | ***P* value** |
| --- | --- | --- | --- | --- |
| **Age（years）** | 33.95 ± 3.75 | 34.59 ± 3.65 | -1.416 | 0.159 |
| **Education leve** |  |  | - | 0.439 |
| Junior high school or below | 13 (5.0) | 6 (7.0) |  |  |
| senior high school | 27 (10.5) | 8 (9.3) |  |  |
| junior college | 64 (24.8) | 15 (17.4) |  |  |
| graduate | 113 (43.8) | 46 (53.5) |  |  |
| Master's degree or above | 41 (15.9) | 11 (12.8) |  |  |
| **Occupation** |  |  | - | 0.780 |
| civil servant | 55 (21.3) | 24 (27.9) |  |  |
| office clerk | 89 (34.5) | 28 (32.6) |  |  |
| merchant | 19 (7.4) | 6 (7) |  |  |
| worker | 11 (4.3) | 4 (4.7) |  |  |
| soldier | 15 (5.8) | 6 (7) |  |  |
| farmer | 13 (5.0) | 5 (5.8) |  |  |
| others | 56 (21.7) | 13 (15.1) |  |  |
| **Marital status** |  |  | - | 0.914 |
| married | 245 (95.0) | 83 (96.5) |  |  |
| discoverture | 7 (2.7) | 2 (2.3) |  |  |
| Divorce | 6 (2.3) | 1 (1.2) |  |  |
| **Household income（RMB）** |  |  | - | 0.890 |
| ＜3000 | 6 (2.3) | 2 (2.3) |  |  |

Supplementary TABLE 1(Continued )

| **Rike factor** | **Non-DOL**  **(*n* = 258)** | **DOL**  **(*n* = 86)** | **Statistic** | ***P* value** |
| --- | --- | --- | --- | --- |
| 3000~5000 | 20 (7.8) | 6 (7.0) |  |  |
| 5001~10000 | 85 (32.9) | 25 (29.1) |  |  |
| ＞10000 | 147 (57.0) | 53 (61.6) |  |  |
| **Residential location** |  |  | 0.180 | 0.671 |
| cities and towns | 218 (84.5) | 71 (82.6) |  |  |
| country | 40 (15.5) | 15 (17.4) |  |  |
| **Number of caregivers** | 1.41 ± 0.66 | 1.52 ± 0.70 | -1.312 | 0.192 |
| **Stressful event** |  |  | - | 0.261 |
| Yes | 2 (0.8) | 2 (2.3) |  |  |
| No | 256 (99.2) | 84 (97.7) |  |  |
| **Breastfeeding knowledge training** |  |  | 1.935 | 0.164 |
| Yes | 157 (60.9) | 45 (52.3) |  |  |
| No | 101 (39.1) | 41 (47.7) |  |  |
| **Gender of the newborn** |  |  | 0.777 | 0.378 |
| Boy | 145 (56.2) | 53 (61.6) |  |  |
| Girl | 113 (43.8) | 33 (38.4) |  |  |
| Breastfeeding experience |  |  | 1.606 | 0.205 |
| Yes | 110 (42.6) | 30 (34.9) |  |  |
| No | 148 (57.4) | 56 (65.1) |  |  |
| Intended feeding method |  |  | 0.195 | 0.658 |
| Breedfeeding | 109 (42.2) | 34 (39.5) |  |  |
| Mixed feeding | 149 (57.8) | 52 (60.5) |  |  |
| **Pre-pregnancy BMI（kg/m^2^）** | 22.58 ± 3.57 | 23.12 ± 4.00 | -1.111 | 0.268 |
| **Pre-delivery BMI（kg/m^2^）** | 26.66 ± 3.38 | 28.11 ± 3.64 | -3.258 | 0.001^*^ |
| **Breastfeeding frequency within**  **24 hours(times)** | 7.14 ± 1.15 | 7.14 ± 1.15 | -0.027 | 0.978 |

Supplementary TABLE 1(Continued )

| **Rike factor** | **Non-DOL**  **(*n* = 258)** | **DOL**  **(*n* = 86)** | **Statistic** | ***P* value** |
| --- | --- | --- | --- | --- |
| **Breastfeeding frequency within**  **24~48 hours(times)** | 7.53 ± 1.09 | 7.24 ± 1.25 | 1.907 | 0.059 |
| **Rooming-in** |  |  | - | 0.098 |
| Yes | 251 (97.3) | 80 (93.0) |  |  |
| No | 7 (2.7) | 6 (7.0) |  |  |
| **Timing of first suckling** |  |  | 2.196 | 0.533 |
| ≤1h | 158 (61.2) | 52 (60.5) |  |  |
| 1~2h | 47 (18.2) | 16 (18.6) |  |  |
| 2~6h | 34 (13.2) | 8 (9.3) |  |  |
| ＞6h | 19 (7.4) | 10 (11.6) |  |  |
| **EPDS score** | 7.03 ± 4.04 | 9.42 ± 3.00 | -5.834 | < 0.001^*^ |
| **SAS score** | 35.51 ± 7.70 | 38.34 ± 7.78 | -2.933 | 0.004^*^ |
| **PSQI score** | 6.45 ± 3.76 | 7.60 ± 3.58 | -2.562 | 0.011^*^ |
| **SSRS score** | 41.95 ± 6.74 | 39.78 ± 6.90 | 2.540 | 0.012^*^ |
| Gestational weeks | 38.67 ± 1.01 | 38.55 ± 1.05 | 0.913 | 0.363 |
| **Parity** |  |  | 1.277 | 0.259 |
| primiparity | 141 (54.7) | 53 (61.6) |  |  |
| multiparity | 117 (45.3) | 33 (38.4) |  |  |
| **Delivery mode** |  |  | 2.422 | 0.298 |
| scheduled cesarean section | 88 (34.1) | 26 (30.2) |  |  |
| emergency cesarean section | 73 (28.3) | 32 (37.2) |  |  |
| eutocia | 97 (37.6) | 28 (32.6) |  |  |
| **Neonatal weight（kg）** | 3.13 ± 0.42 | 3.14 ± 0.45 | -0.155 | 0.877 |
| **formula supplementation** |  |  | 0.045 | 0.832 |
| Yes | 189 (73.3) | 64 (74.4) |  |  |

Supplementary TABLE 1(Continued )

| **Rike factor** | **Non-DOL**  **(*n* = 258)** | **DOL**  **(*n* = 86)** | **Statistic** | ***P* value** |
| --- | --- | --- | --- | --- |
| No | 69 (26.7) | 22 (25.6) |  |  |
| **Limosis OGTT（mmol/L）** | 4.68 ± 0.58 | 4.69 ± 0.48 | -0.222 | 0.825 |
| **1h OGTT（mmol/L）** | 10.12 ± 1.39 | 10.17 ± 1.37 | -0.298 | 0.766 |
| **2h OGTT（mmol/L）** | 8.92 ± 1.38 | 8.61 ± 1.25 | 1.955 | 0.052 |
| **Serum albumin(g/L)** | 34.41 ± 2.36 | 33.56 ± 2.62 | 2.688 | 0.008^*^ |
| **TC（mmol/L）** | 6.58 ± 1.51 | 6.41 ± 1.19 | 1.044 | 0.298 |
| **TG（mmol/L）** | 3.58 ± 1.51 | 3.77 ± 1.81 | -0.870 | 0.386 |
| **HDL-c（mmol/L）** | 1.96 ± 0.48 | 1.95 ± 0.38 | 0.337 | 0.736 |
| **LDL-c（mmol/L）** | 3.74 ± 0.94 | 3.63 ± 0.69 | 1.106 | 0.270 |
| **Neonatal hypoglycemia** |  |  | 2.844 | 0.092 |
| Yes | 37 (14.3) | 19 (22.1) |  |  |
| No | 221 (85.7) | 67 (77.9) |  |  |
| **GDM treatment regimen** |  |  | - | 1.000 |
| Diet and exercise | 252 (97.7) | 84 (97.7) |  |  |
| hypoglycemic agent | 3 (1.2) | 1 (1.2) |  |  |
| insulin | 3 (1.2) | 1 (1.2) |  |  |
| **Labor analgesia** |  |  | 0.503 | 0.478 |
| Yes | 98 (38.0) | 29 (33.7) |  |  |
| No | 160 (62.0) | 57 (66.3) |  |  |
| **Main feeding methods** |  |  | 3.876 | 0.049^*^ |
| Breedfeeding | 51 (19.8) | 9 (10.5) |  |  |
| Mixed feeding | 207 (80.2) | 77 (89.5) |  |  |
| **LATCH score** | 8.62 ± 1.87 | 6.63 ± 1.95 | 8.260 | < 0.001^*^ |
| **Glycemic control during pregnancy** |  |  | 21.578 | < 0.001^*^ |
| Good | 164 (63.6) | 30 (34.9) |  |  |
| Poor | 94 (36.4) | 56 (65.1) |  |  |

Abbreviations: BMI , Body mass index; EPDS, Edinburgh Postnatal Depression Scale; SAS, Self-Rating Anxiety Scale; EPDS, Edinburgh Postnatal Depression Scale; SSRS, Social Support Revalued scale; TC, Total cholesterol ;TG, Triglyceride; HDL-c, High-density lipoprotein cholesterol; LDL-c, Low-density lipoprotein cholesterol ;**P*＜0.05.
